# Children’s mental health hospital throughout COVID-19

**DOI:** 10.1192/j.eurpsy.2021.737

**Published:** 2021-08-13

**Authors:** O. Shchedrinskaya, A. Basova, M. Bebtschuk

**Affiliations:** Science, Moscow State Budgetary Health Care Institution “Scientific and Practical Center for Mental Health of Children and Adolescents named after G.E.Sukhareva of Moscow Health Department, Moscow, Russia, Moscow, Russian Federation

**Keywords:** epidemiology, Child Psychiatry, mental health clinic, psychiatric unit

## Abstract

**Introduction:**

Children with mental health issues are heavily dependent upon the attitude of their caregivers towards disease control and prevention. There is also a high risk of COVID-19 outbreaks in the mental health clinics (hospitals) for children.

**Objectives:**

Children with mental health issues are heavily dependent upon the attitude of their caregivers towards disease control and prevention. There is also a high risk of COVID-19 outbreaks in the mental health clinics (hospitals) for children.

**Methods:**

The data from the Municipal Department of Health and Medical records of patients who were treated at the clinic from January to June in 2019 were compared. Statistical processing was carried out using the Chi-Square for 2 x 2 Contingency Table method.

**Results:**

The study demonstrated the statistically significant difference in the types of mental health conditions that require more attention and in-patient emergency treatment options during the pandemic, including decompensation, exacerbation or manifestation of endogenous diseases, anorexia nervosa and suicidal manifestations. To prevent the spread of infection, a specific separate clinical unit was created for patients with severe mental health disorders and symptoms of COVID-19. Additionally, multiple changes were implemented in treatment protocols, staff duties and interactions with the patients’ caregivers.

**Conclusions:**

There was a higher demand for in-patient emergency treatment for children with severe mental health disorders in 2020, as compared to 2019. Timely introduced anti-epidemic measures made it possible to avoid outbreaks of COVID-19 in the children’s psychiatric hospital.

